# Cervical Cancer Prevention and Treatment Disparities Among Native Hawaiian and Pacific Islanders: A Systematic Review and Meta‐Analysis

**DOI:** 10.1002/jso.70200

**Published:** 2026-01-28

**Authors:** Antoinette T. Nguyen, Emily D. Duckworth, Rena A. Li, Tarifa H. Adam, Robert D. Galiano

**Affiliations:** ^1^ University of Rochester School of Medicine and Dentistry Rochester New York USA; ^2^ University of South Carolina School of Medicine Greenville Greenville South Carolina USA; ^3^ Northwestern University Feinberg School of Medicine Chicago Illinois USA

**Keywords:** cervical cancer, health disparities, HPV vaccination, native Hawaiian, Pacific Islander, Pap testing

## Abstract

**Background:**

Native Hawaiian and Pacific Islander (NHPI) populations face significant disparities in cervical cancer prevention and treatment. This systematic review and meta‐analysis examines cervical cancer prevention metrics, treatment disparities, and effective interventions among NHPI populations.

**Methods:**

Following PRISMA guidelines, we systematically searched PubMed, Scopus, and Embase for studies published between 2000 and 2024 that reported cervical cancer prevention metrics in NHPI populations. Eligible studies included quantitative and qualitative designs with NHPI‐specific or disaggregated data. Pap testing and HPV vaccination rates were pooled using a random‐effects meta‐analysis. Narrative synthesis summarized findings from studies unsuitable for meta‐analysis.

**Results:**

A total of 27 studies were included. The pooled Pap testing rate was 62% (95% CI: 46%–75%), with substantial heterogeneity (I² = 98.7%). The pooled HPV vaccine initiation rate was 25% (95% CI: 16%–37%; I² = 84.3%). Barriers included limited healthcare access, lack of physician recommendations, cultural stigma, and geographic isolation. Effective interventions, such as culturally tailored educational materials and community‐based participatory approaches, demonstrated improved screening and vaccination rates. NHPI patients were less likely to receive timely and guideline‐concordant cervical cancer treatment and had higher rates of late‐stage diagnoses and mortality.

**Conclusions:**

NHPI populations face persistent cervical cancer prevention and treatment disparities. Culturally tailored interventions and policies addressing systemic barriers are critical to reducing these inequities. Future research should focus on longitudinal studies and scalable interventions to improve outcomes in NHPI communities.

## 1 Introduction

Cervical cancer remains a significant public health concern, disproportionately affecting underserved and minority populations worldwide [1]. While rates of cervical cancer incidence and mortality have declined in many high‐income countries due to the widespread implementation of Pap testing and HPV vaccination, persistent disparities exist among specific racial and ethnic groups in the United States [2, 3, 4]. Native Hawaiian and Pacific Islander (NHPI) populations represent a particularly vulnerable group, with unique cultural, geographic, and systemic barriers contributing to lower rates of cervical cancer prevention, delayed treatment, and worse outcomes [5].

Despite their recognized vulnerability, NHPI populations are often underrepresented in research, with data frequently aggregated under broader Asian American categories [6]. This lack of disaggregated data obscures the disparities faced by NHPI communities, which differ substantially from other racial and ethnic groups. For instance, NHPI women are less likely to receive Pap testing or HPV vaccination compared to non‐Hispanic White (NHW) women, and they experience higher rates of late‐stage cervical cancer diagnosis and mortality [7]. Compounding these challenges are systemic issues such as geographic isolation, limited healthcare access, and cultural stigma, which further hinder efforts to address these disparities.

Efforts to improve cervical cancer prevention and treatment outcomes for NHPI populations require a nuanced understanding of their unique barriers and facilitators [8]. Culturally tailored interventions, such as community‐based participatory research (CBPR) and educational campaigns, have shown promise in addressing some of these challenges [9]. However, significant gaps remain in the literature regarding the effectiveness of these strategies and their scalability across diverse NHPI subgroups.

This systematic review and meta‐analysis aims to comprehensively evaluate the current state of cervical cancer prevention and treatment among NHPI populations. Specifically, it examines rates of Pap testing and HPV vaccination, explores disparities in treatment and outcomes, and synthesizes evidence on effective interventions. By highlighting key findings and areas for improvement, this study seeks to inform targeted public health strategies to reduce cervical cancer disparities in this underserved population.

## 2 Methods

**FIGURE 1:**
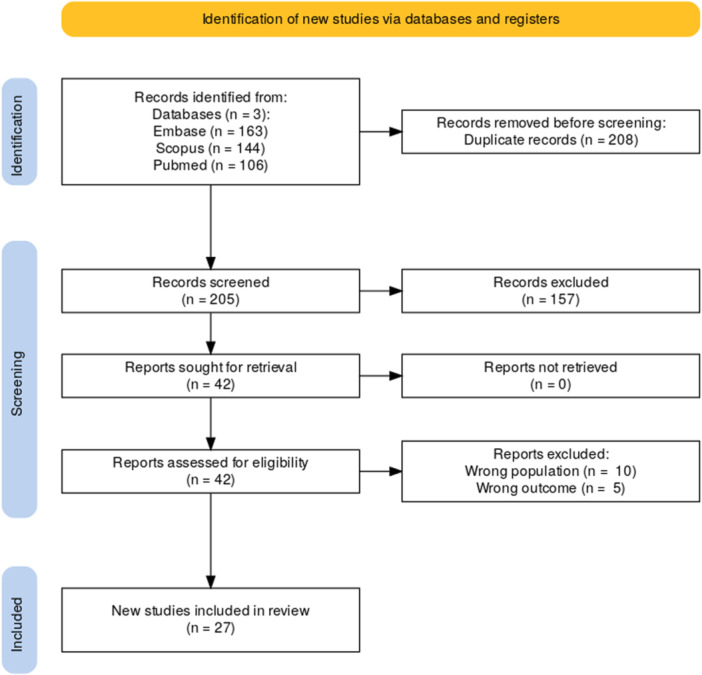
PRISMA diagram. Flow diagram of study selection according to PRISMA guidelines.

This systematic review and meta‐analysis was conducted following PRISMA (Preferred Reporting Items for Systematic Reviews and Meta‐Analyses) guidelines to ensure transparency and rigor. The protocol was registered with PROSPERO (International Prospective Register of Systematic Reviews) under registration number CRD42024628364. A comprehensive search was performed across three databases—PubMed, Scopus, and Embase—to identify studies reporting cervical cancer prevention metrics, including Pap testing rates, HPV vaccination rates, and treatment outcomes among NHPI populations (Figure 1).

Searches were limited to articles published in English from January 2000 to December 2024. In PubMed, terms such as (“Cervical Cancer” [MeSH] OR “Pap Smear” [MeSH] OR “HPV Vaccines” [MeSH]) AND (“Native Hawaiian” OR “Pacific Islander” OR “NHPI” OR “Indigenous Pacific” OR “Polynesian”) were used, while similar strings adapted for Scopus and Embase ensured comprehensive coverage.

Studies were eligible for inclusion if they reported Pap testing or HPV vaccination metrics for NHPI populations or provided disaggregated data for NHPI subgroups. Both quantitative and qualitative study designs, including cross‐sectional, cohort, and randomized controlled trials (RCTs), were considered. Studies published as abstracts, commentaries, or letters without primary data and those that aggregated NHPI populations with other groups without disaggregation were excluded.

Two independent reviewers screened titles and abstracts, followed by full‐text reviews of potentially relevant studies. Any disagreements were resolved through discussion or consultation with a third reviewer. Data extraction was performed using a standardized form to collect information on study characteristics (e.g., author, year, study design, sample size), population demographics, and outcomes such as Pap testing rates, HPV vaccine initiation/completion rates, and treatment metrics.

The quality of included studies was assessed using validated tools appropriate to each study design: the Joanna Briggs Institute (JBI) checklist for cross‐sectional studies, the Newcastle‐Ottawa Scale for cohort studies, and the Cochrane Risk of Bias Tool for RCTs (Table 1). Meta‐analyses were performed for Pap testing and HPV vaccine initiation rates using a random‐effects model to account for variability across studies. Heterogeneity was assessed using the I² statistic and Cochran's Q test. For studies unsuitable for meta‐analysis, a narrative synthesis was conducted, highlighting key findings and contextual insights. All analyses were conducted using the meta package in R software. Sensitivity analyses and publication bias assessments were not pursued due to limited number of eligible studies with necessary race and ethnicity data. While these results would positively impact the robustness of our analyses, limitations in data availability prohibit our ability to broaden our report.

**TABLE 1:** Risk of bias.

Author et al.	Year	Study design	Selection bias	Measurement bias	Confounding bias	Reporting bias	Overall risk of bias
Lee et al.	2024	Retrospective cohort	Low	Moderate	Moderate	Low	Moderate
McDaniel et al.	2021	Cross‐sectional (BRFSS)	Moderate	Moderate	Moderate	Low	Moderate
Kepka et al.	2018	Community‐based survey	Moderate	High	Moderate	High	High
Medina et al.	2021	Population‐based mortality	Low	Low	Low	Low	Low
Dela Cruz et al.	2020	Cross‐sectional	Moderate	Moderate	Moderate	Low	Moderate
Sitler et al.	2023	Retrospective chart review	Moderate	Moderate	Moderate	Low	Moderate
Dela Cruz et al.	2017	Qualitative study	High	High	Moderate	High	High
Shing et al.	2023	Retrospective registry analysis	Low	Low	Low	Low	Low
Mouttapa et al.	2023	Cross‐sectional survey	Moderate	High	Moderate	High	High
Gopalani et al.	2021	Cross‐sectional survey (NHIS)	Low	Low	Moderate	Low	Low
Wong et al.	2010	Qualitative interviews	High	High	High	High	High
Fok et al.	2023	Semi‐structured interviews	High	High	High	High	High
Schisler et al.	2018	Retrospective cohort	Moderate	Moderate	Moderate	Low	Moderate
Tanjasiri et al.	2019	Randomized community trial	Low	Low	Low	Low	Low
Ho et al.	2024	Retrospective cohort	Low	Moderate	Moderate	Low	Moderate
DiStefano et al.	2012	Community‐based participatory	High	High	Moderate	High	High
Chen et al.	2004	Cross‐sectional survey	Moderate	Moderate	Moderate	Moderate	Moderate
Mouttapa et al.	2016	Cross‐sectional survey	Moderate	Moderate	Moderate	Moderate	Moderate
Tanjasiri et al.	2012	Cross‐sectional survey	Moderate	Moderate	Moderate	Moderate	Moderate
Aitaoto et al.	2009	Qualitative focus groups	High	High	High	High	High
Robison et al.	2002	Retrospective cohort	Moderate	Moderate	Moderate	Moderate	Moderate
Weiss et al.	2016	Cross‐sectional survey	Moderate	Moderate	Moderate	Moderate	Moderate
Novinson et al.	2017	Pre‐post educational study	Moderate	Moderate	Moderate	Moderate	Moderate
Gotay et al.	2000	Community intervention	Moderate	Moderate	Moderate	Moderate	Moderate
Tran et al.	2013	Cross‐sectional survey	Moderate	Moderate	Moderate	Moderate	Moderate
Tanjasiri et al.	2015	Randomized community trial	Low	Low	Low	Low	Low

*Note:* Summary of risk of bias domains for all studies using appropriate tools based on study design.

## 3 Results

A comprehensive narrative synthesis was conducted, incorporating all 27 studies that addressed cervical cancer prevention metrics such as Pap testing rates, HPV vaccination uptake, and treatment outcomes. These studies highlight persistent disparities in cervical cancer prevention and care among Native Hawaiian and Pacific Islander (NHPI) populations and emphasize the importance of targeted, culturally tailored interventions (Table 2).

**TABLE 2:** Summary of included studies.

Study	Study design	Total participants	Number of NHPI participants	Cervical cancer metric	Statistical analyses	Key findings
Lee et al., 2024	Retrospective cohort study using NCDB data	48 116	171	Guideline‐concordant care (treatment timing, brachytherapy, chemotherapy)	NHPI patients were less likely to receive care at high‐volume facilities (22%) compared to Hispanic/Latine patients (43%) and had the longest delays in treatment initiation (39% starting after 8 weeks, vs. 24% for NHW patients). Median overall survival (OS) for NH/PI patients was 60 months.	NHPI patients experience significant disparities in cervical cancer treatment compared to NHW and Hispanic/Latine groups, with lower access to high‐volume facilities and greater delays in care, which may contribute to worse outcomes. Tailored interventions are necessary to address these barriers.
McDaniel et al., 2021	Cross‐sectional study (BRFSS)	538 218	~1184	Screening behavior (Pap test)	NHPI women were 66% less likely to have received a Pap test compared to White women (adjusted OR: 0.339, 95% CI: 0.249–0.462). Overall Pap test rates improved slightly for NHPI women from 73.95% in 2014 to 82.98% in 2018.	NHPI women consistently showed lower Pap test screening rates compared to White women. The study emphasizes systemic disparities, including access barriers, and highlights a need for equity‐focused interventions to address these gaps in care.
Kepka et al., 2018	Community‐based participatory research (survey study)	228	70 (30.7%)	HPV vaccination (receipt, intent, barriers)	NHPI caregivers had the lowest HPV vaccination rate for their children (10.6%, compared to 47.1% for AI/AN and 29.8% for Hispanic/Latino). Key barriers cited included lack of knowledge (52%), cost (18%), and concerns about side effects (18%).	NHPI caregivers reported the lowest HPV vaccination rates among racial/ethnic groups in the study. Barriers included lack of knowledge, cost concerns, and misconceptions about side effects, signaling the need for targeted, community‐specific interventions.
Medina et al., 2021	Population‐based mortality analysis	260 914 cancer deaths	1447 NHPI (0.6%)	Cancer mortality (cervical and others)	NHPI women had 7.1 times higher cervical cancer mortality (MRR: 7.1, 95% CI: 4.8–9.4) compared to NHW women. NHPI women also had disproportionately high mortality rates for breast and endometrial cancers (MRR: 1.3 and 3.3, respectively).	NHPI women experience significant disparities in cervical cancer mortality, with rates much higher than NHW women. The findings emphasize the need for targeted interventions to reduce cervical cancer mortality and improve screening in this population.
Dela Cruz et al., 2020	Population‐based, cross‐sectional survey	799	189 (23.7%)	HPV vaccination (uptake, barriers)	35.2% of girls and 18.8% of boys (ages 11–18) completed all three doses of the HPV vaccine. NHPI parents reported lower completion rates for daughters (37.9%) and sons (18.0%) compared to Japanese parents (40.8% and 22.5%, respectively).	NHPI parents in Hawaii face unique barriers to HPV vaccination, including lack of physician recommendation and limited knowledge. Despite lower vaccination rates, physician recommendation and education are strong motivators to improve uptake.
Sitler et al., 2023	Retrospective chart review	214	67 (31.3%)	Standard‐of‐care (SOC) radiation therapy for LACC	71.6% of NHPI patients received brachytherapy, significantly higher than the U.S. average of 49.5% (*p* < 0.001). Similarly, 62.7% received SOC treatment (EBRT, brachytherapy, and chemotherapy) compared to 44.3% nationwide (*p* < 0.001).	NHPI women with LACC treated at TAMC as part of the PIHCP had significantly higher adherence to SOC treatments compared to the U.S. population. The findings highlight the effectiveness of the PIHCP in improving care for underserved populations.
Dela Cruz et al., 2017	Qualitative study (interviews)	20 parents (30 children)	five parents identified as Native Hawaiian (25%)	HPV vaccination motivators, barriers	Of the 30 children, 33% were fully vaccinated, 27% partially vaccinated, and 40% unvaccinated. Parental knowledge, physician recommendations, and culturally appropriate educational materials were critical motivators to increase uptake. Barriers included concerns about timing and misconceptions about HPV vaccine implications.	NHPI parents valued physician recommendations and culturally tailored brochures but faced significant barriers, including lack of knowledge and vaccine stigma. Recommendations include using culturally relevant materials and targeted education campaigns to address hesitancy.
Shing et al., 2023	Retrospective registry analysis (SEER database)	81 million person‐years (women)	~7695 (0.0095%)	HPV‐associated cancer incidence	NHPI women had a cervical cancer incidence rate of 9.5 per 100,000 (95% CI: 8.6–10.5), higher than aggregated Asian Americans (7.4) and non‐Hispanic White women (7.4). NHPI women did not experience statistically significant declines in cervical cancer incidence over time.	NHPI women have disproportionately high cervical cancer incidence rates, with a lack of decline over decades. These findings highlight the critical need for disaggregated data and targeted interventions to address cervical cancer disparities in this group.
Mouttapa et al., 2023	Cross‐sectional survey	148	148 (100%)	HPV awareness and vaccination	64.2% had heard of HPV, and 59.5% were aware of the HPV vaccine. Only 30.3% of women under 40 years had received the vaccine. Acculturation to American culture increased awareness of HPV but reduced willingness to vaccinate children (AOR = 0.82, *p* = 0.03).	Pacific Islander women in Southern California have moderate awareness of HPV and its vaccine, but low vaccination rates. Interventions should address cultural nuances and misconceptions, emphasizing physician recommendations and tailored education.
Gopalani et al., 2021	Cross‐sectional survey (NHIS)	1204	1204 (100%)	HPV vaccination rates	24.9% of NHPI adults aged 18–26 initiated HPV vaccination, and 11.5% completed the series. Women had 5.4 times higher odds of initiating the vaccine compared to men (OR = 5.4, 95% CI: 2.8–10.4). Vaccination rates were lower compared to other racial groups.	HPV vaccination rates among NHPI adults are low compared to other racial groups. Gender disparities are notable, with significantly higher uptake among women. Interventions targeting cultural and systemic barriers are necessary to improve vaccination rates.
Wong et al., 2010	Qualitative study (key informant interviews)	10	10 (100%)	Cervical cancer knowledge, prevention, and screening	90% of Chuukese women had limited knowledge about cervical cancer and no awareness of HPV or the HPV vaccine. Pap smears were viewed as shameful, with cultural and privacy concerns being major barriers. All participants agreed cervical cancer is curable if detected early.	Chuukese women in Hawai'i face significant cultural barriers to cervical cancer prevention, with low awareness of HPV and Pap smears. Education tailored to their cultural context, led by women, could improve screening rates and cancer prevention efforts.
Fok et al., 2023	Qualitative study (semi‐structured interviews)	15 parents	15 parents (100%)	HPV vaccination barriers and motivators	66.7% of parents expressed hesitancy toward HPV vaccination due to unfamiliarity (67%), concerns about reproductive health (20%), and cultural taboos (40%). Medical professional recommendations increased vaccination uptake in 85% of cases.	NHPI parents in Orange County face cultural and knowledge‐based barriers to HPV vaccination. Tailored, culturally relevant education and trusted healthcare provider recommendations are critical to increasing HPV vaccination rates.
Schisler et al., 2018	Retrospective cohort study	35	35 (100%)	HPV genotypes in cervical cancer patients	HPV DNA was identified in 71% of samples, with the most common genotypes being HPV 16 (24%), 45 (24%), and 52 (24%). Only 28% of cases were preventable with the quadrivalent vaccine, but the nonavalent vaccine could protect up to 88% of cases.	NHPI cervical cancer patients had high rates of regional and advanced‐stage disease at presentation. The study underscores the potential benefit of broader HPV vaccination coverage in this population using the nonavalent vaccine.
Tanjasiri et al., 2019	Randomized community trial	591 women, 416 men	591 (100%)	Pap testing uptake	55.4% of noncompliant women in the intervention group scheduled a Pap test, and 51.4% received a Pap test within 6 months, compared to 40.2% and 34.9% in the comparison group, respectively. Social support interventions showed significant effects on behavior but not on attitudes.	Social support‐focused community interventions significantly improved Pap testing rates among Pacific Islander women in Southern California, highlighting the effectiveness of culturally tailored approaches for collectivist communities.
Ho et al., 2024	Retrospective cohort study (NCDB analysis)	122 926	519 (4.2% of total patients were NHPI)	Stage at presentation	NHPI women were 1.58 times more likely to present with metastatic disease compared to NHW patients (aOR: 1.58, 95% CI: 1.21–2.06, *p* = 0.001). Disaggregated data revealed that Pacific Islanders were 3.44 times more likely to present with advanced disease than Chinese Americans.	NHPI women experience significant disparities in stage at presentation for cervical cancer, with Pacific Islanders facing the highest odds of late‐stage presentation. Structural barriers and socioeconomic factors contribute to disparities.
DiStefano et al., 2012	Inductive community‐based participatory research	95	95 (100%)	HPV risk and prevention	Limited knowledge of HPV, with most participants unaware of its link to cervical cancer. Gendered cultural norms and restricted intergenerational communication were significant barriers to screening and vaccination. Risk behaviors included inconsistent safer sex practices.	Cultural barriers, including gender norms and family shame, significantly impede HPV prevention in Pacific Islander young adults. Recommendations include culturally tailored interventions and engaging community leaders to address stigma and barriers.
Chen et al., 2004	Cross‐sectional population survey (Los Angeles County Health Survey)	8353 adults (4975 women)	14 NHPI women (3.6% of AAPI women)	Pap smear rates and cervical cancer screening	Pacific Islanders had a Pap smear rate of 72%, significantly lower than White women (81%) but higher than some other Asian subgroups such as Chinese (56%) and Korean (65%). After adjusting for covariates, NHPI women still had lower odds of screening compared to Whites (OR = 0.72, *p* = 0.18).	NHPI women face lower cervical cancer screening rates compared to Whites, highlighting the need for culturally sensitive interventions and better access to healthcare for Pacific Islanders in the United States.
Lee et al., 2024	Retrospective cohort study (NCDB)	48 116	171 (0.4%)	Guideline‐concordant care	NHPI patients had lower rates of receiving treatment at high‐volume facilities (22% vs. 33% for AAPI overall, *p* < 0.001) and higher rates of treatment initiation delays (39% vs. 31% for AAPI overall, *p* < 0.001). Median overall survival for NH/PI was 60 months, similar to non‐Hispanic White patients.	NHPI patients with cervical cancer faced systemic barriers, including low access to high‐volume facilities and significant treatment initiation delays, potentially contributing to disparities in care. Despite these, survival outcomes were similar to NHW patients.
Mouttapa et al., 2016	Cross‐sectional survey	585	585 (100%)	Pap test rates and social support	52.3% of women had a Pap test within 3 years. Pap testing rates were significantly higher among those with strong perceived emotional support (*p* < 0.05) and informational support (*p* < 0.05). Tongan women had significantly lower Pap testing rates compared to Samoan and Chamorro women (31.4% vs. 61.3%).	Social support from husbands or partners, particularly emotional and informational support, significantly influenced Pap testing rates among Pacific Islander women. Interventions targeting family‐based support could increase screening uptake.
Tanjasiri et al., 2012	Cross‐sectional survey	418	418 (100%)	Pap test rates and predictors	66% of Chamorro women had received a Pap test within the last 2 years, below the U.S. average (72%). Predictors included age (younger women more likely), health insurance (OR = 4.41, *p* = 0.03), and knowledge of recommended screening frequency (OR = 2.49, *p* = 0.01).	Chamorro women in California face lower cervical cancer screening rates, driven by limited knowledge and access barriers. Culturally tailored interventions and improving health insurance coverage could enhance screening rates.
Aitaoto et al., 2009	Qualitative focus groups and interviews	33 focus group participants, nine providers	33 (15 Native Hawaiian, 12 Chuukese/Marshallese, six Filipina)	Pap test and cervical cancer screening barriers	73% of participants had received a Pap test, but only 33% of Chuukese/Marshallese women had access compared to 93% of Native Hawaiians. Major barriers included cultural beliefs, lack of transportation, competing priorities, and fear of bad news. Effective strategies included one‐on‐one education, lay educators, and culturally tailored outreach campaigns.	Native Hawaiian, Pacific Islander, and Filipina women face systemic and cultural barriers to cervical cancer screening. Tailored community and culturally relevant outreach, supported by lay educators, significantly improves screening participation.
Robison et al., 2002	Retrospective cohort study (U.S. Military Health Care System)	153	74 (48%)	Survival rates by ethnicity	Pacific Islanders (PI) had a significantly lower 5‐year survival rate (32%) compared to non‐Pacific Islander (71%) and Continental U.S. (76%) patients (*p* < 0.001). PI women presented with more advanced‐stage disease (74% stages II‐IV) and larger tumors (5.75 cm vs. 1.86 cm, *p* < 0.001).	PI women with cervical cancer face significantly worse survival outcomes due to advanced‐stage diagnosis and metastatic disease. Barriers include lack of routine screening and access to care, highlighting the need for systemic interventions.
Weiss et al., 2016	Cross‐sectional survey	585	585 (100%)	Pap test rates and decision‐making	53.2% of women had Pap testing in the past 3 years. Pap testing was significantly associated with higher utility scores for detecting cervical cancer early (AOR = 1.10, 95% CI: 1.04–1.17, *p* = 0.001), peace of mind, and protecting family.	Pacific Islander women in Southern California face barriers to Pap testing, including cultural and emotional factors. Positive anticipated consequences, such as peace of mind and family protection, strongly influence decision‐making.
Novinson et al., 2017	Pre‐post educational intervention	326 women, 29 providers	326 women (100% A/NH/PI), 27 providers (93% A/NH/PI)	Knowledge of HPV risks and cervical cancer screening	Public knowledge that HPV causes cervical, vulvar, and vaginal cancers increased from 4.9% to 51.4% (*p* < 0.0001). Smoking as a risk factor for cervical cancer increased awareness from 53.8% to 98.7% (*p* < 0.0001). HPV vaccination intentions among eligible participants increased from 81% to 95% (*p* = 0.0039).	Culturally tailored education effectively increased awareness of cervical cancer risks and prevention measures among A/NH/PI women in USAPIJ, addressing a critical gap in health knowledge in underserved regions.
Gotay et al., 2000	Community‐based intervention study	678	678 (100%)	Pap test rates and screening adherence	Women in the intervention community reported significantly increased Pap screening adherence (67% vs. 59% at baseline, *p* = 0.02) compared to no significant changes in the control community (64% vs. 63%, *p* > 0.05). Women exposed to the Kokua Groups were more likely to encourage others to undergo screening (42% vs. 28%, *p* = 0.02).	A culturally tailored intervention leveraging social support networks (Kokua Groups) effectively improved Pap screening adherence and promoted health‐seeking behaviors in Native Hawaiian women, with diffusion of knowledge enhancing community impact.
Tran et al., 2013	Cross‐sectional survey (CBPR)	157	157 (100%)	Screening compliance rates	59.2% of participants were compliant with age‐specific breast and cervical cancer screenings. Knowledge of correct screening procedures (AOR = 2.43, *p* = 0.049), seeking health advice from doctors (AOR = 12.12, *p* = 0.033), and use of internet medical sites (AOR = 5.39, *p* = 0.001) were significant predictors of compliance.	Screening compliance among Native Hawaiians in Southern California was influenced by access to health information and sources. Key barriers included lack of knowledge and cultural factors, emphasizing the need for culturally sensitive interventions.
Tanjasiri et al., 2015	Randomized community trial	473 women, 419 men	473 women (100% NHPI)	Pap testing rates and retention	71% of AAPI women had received a Pap test within 3 years compared to the U.S. average of 82%. Social support interventions significantly improved intentions and behaviors. Retention rates at 6 months were 63.5% for women and 60.5% for men.	CBPR strategies tailored to Pacific Islander communities were effective for improving Pap test education and participation but faced challenges in retention. Addressing cultural and logistical barriers was key to sustained engagement.

*Note:* Summary of study design, methodology, population characteristics, statistical analysis, and key findings.

### 3.1 Pap Testing Rates

Pap testing rates were generally suboptimal across studies, with significant variability. Chen et al. reported that 72% of NHPI women in Los Angeles County had undergone a Pap test, a rate lower than non‐Hispanic White (NHW) women (81%) but higher than some other Asian subgroups, such as Chinese (56%) and Korean (65%) [10]. Similarly, McDaniel et al. observed that NHPI women were significantly less likely to have received a Pap test compared to NHW women (adjusted OR: 0.339) [11]. Despite improvements in Pap testing rates among NHPI women between 2014 and 2018 (from 73.95% to 82.98%), disparities persisted compared to other groups.

Aitaoto et al. highlighted the barriers specific to Chuukese and Marshallese women in Hawaii, where only 33% reported access to Pap tests compared to 93% of Native Hawaiian women [12]. Weiss et al., Tanjasiri et al. (2012), and Mouttapa (2016) found that emotional and informational support from family members significantly influenced Pap testing behavior, with Tongan women reporting disproportionately lower screening rates compared to other Pacific Islander subgroups [13, 14, 15].

Interventions have been shown to improve Pap testing rates in specific NHPI communities. Tanjasiri et al. (2019) demonstrated that community‐based social support interventions significantly increased Pap testing rates, from 40.2% in the control group to 55.4% in the intervention group [16]. Similarly, Tran et al. found that women who sought advice from healthcare providers were significantly more likely to complete screening (AOR = 12.12, *p* = 0.033) [17]. Novinson et al. found that an educational intervention in the US‐Associated Pacific Island Jurisdictions increased knowledge about cervical cancer and HPV risks, which likely translated into improved screening behaviors [18].

### 3.2 HPV Vaccination Rates

HPV vaccination rates were consistently low across NHPI populations, with significant barriers identified in multiple studies. Gopalani et al. reported an HPV vaccine initiation rate of 24.9% among NHPI adults aged 18–26, with completion rates as low as 11.5% [19]. Gender disparities were evident, with women having 5.4 times higher odds of initiating vaccination than men. Dela Cruz et al. (2020) found that 35.2% of NHPI adolescents in Hawaii initiated HPV vaccination, but barriers included a lack of physician recommendations and limited knowledge [20].

Cultural stigma and misconceptions about the vaccine were recurring themes. Fok et al. found that 40% of NHPI parents associated the vaccine with taboo topics, while Mouttapa et al. (2023) highlighted concerns about reproductive health and vaccine side effects [21, 22]. Schisler et al. added an important dimension by identifying that the nonavalent HPV vaccine could protect against up to 88% of HPV‐related cancers in NHPI populations, underscoring the potential impact of improving vaccination rates [23].

Interventions have shown promise in addressing these barriers. Dela Cruz et al. (2017) reported that culturally tailored educational materials and physician recommendations significantly improved parental willingness to vaccinate their children [24]. Similarly, Novinson et al. demonstrated that targeted educational campaigns increased HPV vaccination intentions from 81% to 95% (*p* = 0.0039) in underserved NHPI communities [18].

### 3.3 Cervical Cancer Treatment and Outcomes

Disparities in cervical cancer treatment and outcomes were consistently documented. Lee et al. found that NHPI patients with locally advanced cervical cancer were less likely to receive care at high‐volume facilities (22% compared to 33% for Asian Americans overall) and experienced the longest delays in treatment initiation (39% starting after 8 weeks) [25]. Sitler et al. reported that NHPI patients treated through the Pacific Island Health Care Project (PIHCP) had higher adherence to standard‐of‐care radiation therapy (71.6%) compared to the U.S. average (49.5%) [26].

Disaggregated data further highlighted disparities among NHPI subgroups. Ho et al. found that NHPI women were 1.58 times more likely to present with metastatic cervical cancer compared to NHW women (aOR: 1.58, *p* = 0.001) [27]. Pacific Islanders had disproportionately high rates of advanced‐stage disease. Robison et al. also found that Pacific Islanders diagnosed with cervical cancer had significantly worse 5‐year survival rates (32%) compared to NHW women (71%) [28].

### 3.4 Barriers and Facilitators

Barriers to cervical cancer prevention in NHPI populations included systemic, cultural, and individual factors. Systemic barriers include limited healthcare access, physician recommendations, and geographic isolation, as seen in studies like Lee et al. and Sitler et al. [25, 26]. Cultural barriers include misconceptions about cervical cancer and HPV, as well as cultural stigma, which were noted in studies such as Fok et al. and Schisler et al. [21, 23]. However, some studies presented interventions to combat these barriers, including community‐based participatory research (CBPR) approaches, culturally tailored educational interventions, and strong social support networks, that were effective in addressing barriers. Studies such as Tanjasiri et al. (2015) and Gotay et al. demonstrated the value of leveraging culturally relevant outreach to improve screening and vaccination rates [29, 30].

### 3.5 Meta‐Analysis of Pap Testing Rates

**FIGURE 2:**
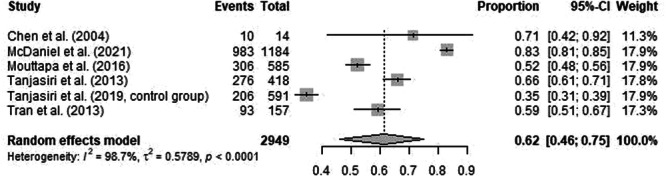
Forest plot of pap testing rates meta‐analysis. Forest plot showing pooled odds ratios for Pap testing rates.

**FIGURE 3:**
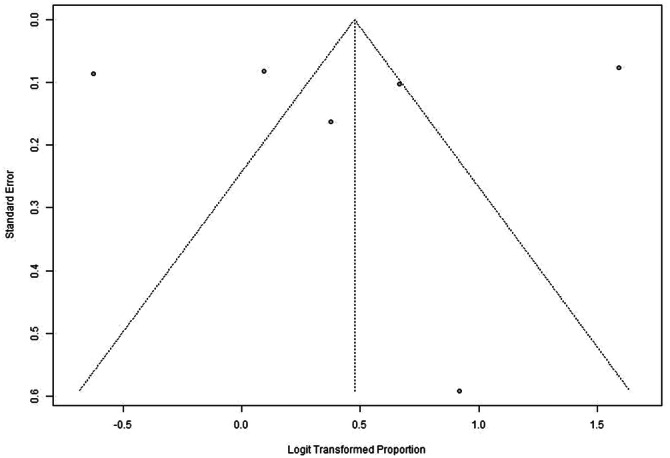
Funnel plot of pap testing rates meta‐analysis. Funnel plot assessing Pap testing rates among studies included in the meta‐analysis.

The meta‐analysis included six studies that reported Pap testing rates among NHPI populations. These studies varied in design and population sizes, ranging from 14 participants in Chen et al. to 1184 participants in McDaniel et al. [10, 11]. The pooled estimate of Pap testing rates was 62% (95% CI: 46%–75%) using a random‐effects model, reflecting moderate uptake among NHPI populations. Significant heterogeneity was observed (I² = 98.7%, *p* < 0.0001) (Figures 2 and 3).

### 3.6 Meta‐Analysis of HPV Vaccine Initiation Rates

**FIGURE 4:**
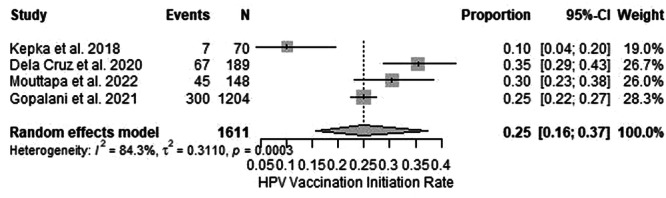
Forest plot of HPV vaccination rates meta‐analysis. Forest plot showing pooled odds ratios for HPV vaccination rates.

**FIGURE 5:**
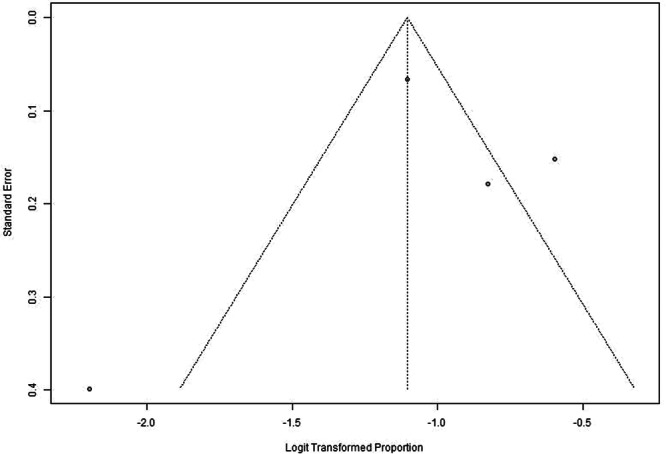
Funnel plot of HPV vaccination rates meta‐analysis. Funnel plot assessing HPV vaccination rates among studies included in the meta‐analysis.

The meta‐analysis included four studies reporting HPV vaccine initiation rates among NHPI populations. These studies encompassed a range of geographic and demographic contexts, with sample sizes varying from 70 participants in Kepka et al. to 1204 participants in Gopalani et al. [19, 31]. The pooled estimate for HPV vaccine initiation was 25% (95% CI: 16%–37%) under the random‐effects model, reflecting significant room for improvement in vaccine uptake among NHPI populations. High heterogeneity was observed (I² = 84.3%, *p* < 0.001), indicating considerable variability across studies (Figures 4 and 5).

## 4 Discussion

The findings of this comprehensive synthesis and meta‐analysis elucidate the profound disparities in cervical cancer prevention, vaccination, and treatment among NHPI populations. While the results affirm the existence of systemic, cultural, and logistical barriers, they also reveal nuanced interplays between these factors, warranting a deeper analysis to inform actionable strategies.

### 4.1 Pap Testing Rates

The pooled Pap testing rate of 62% among NHPI populations falls significantly short of the U.S. national average (~80%), underscoring persistent gaps in preventive care [32]. This shortfall not only highlights inequities but also signals a broader structural issue in the integration of NHPI populations into preventive healthcare systems. Despite some gains in national datasets such as the Behavioral Risk Factor Surveillance System (BRFSS) reported by McDaniel et al., the variability across studies (e.g., 40.2% in Tanjasiri et al. (2019) vs. 72% in Chen et al.) suggests that geographic and cultural contexts critically shape outcomes [10, 11, 16].

Barriers to Pap testing among NHPI populations often intersect with structural and cultural determinants. Geographic isolation in Pacific territories limits access to screening facilities, exacerbated by economic disparities that hinder travel to urban centers [25]. Additionally, cultural perceptions of modesty, family shame, and mistrust of healthcare providers further impede screening, as seen in Chuukese women, whose Pap testing rate was as low as 33% [12]. These barriers are distinct from those seen in other racial groups, where logistical and socioeconomic challenges often dominate; for example, African American women cite financial and time constraints more frequently than cultural stigma as impediments to screening [33].

The success of community‐based participatory research (CBPR) approaches and culturally tailored interventions in improving Pap testing rates highlights the importance of culturally congruent strategies. For example, Tanjasiri et al. (2019) demonstrated a 15% increase in screening rates using social support networks, while Tran et al. showed that direct advice from healthcare providers significantly increased uptake [15, 16]. These findings align with interventions in other underserved populations, such as the use of promotoras (lay health workers) in Hispanic communities, emphasizing the transferability of CBPR principles across cultural contexts [34].

### 4.2 HPV Vaccination Rates

HPV vaccination rates among NHPI populations are alarmingly low, with a pooled initiation rate of 25%. This figure contrasts starkly with national averages (~50% for initiation in adults aged 18–26 and ~60% among adolescents). The disparities become even more pronounced when compared to Hispanic and African American adolescents, whose vaccination rates have shown steady improvement due to targeted public health campaigns [35]. The NHPI‐specific gap, therefore, underscores a lack of focused interventions addressing unique cultural and systemic barriers.

The findings from Fok et al. and Mouttapa et al. (2023) illuminate how deeply rooted cultural factors exacerbate these disparities [21, 22]. Misconceptions linking HPV vaccination to promiscuity, coupled with fears of vaccine‐induced infertility, reflect broader cultural taboos around reproductive health in NHPI communities. These barriers are less frequently observed in other minority groups, where concerns tend to center around cost and access rather than cultural stigma [36]. This suggests that interventions for NHPI populations must go beyond generic education campaigns to directly address culturally specific fears and misconceptions.

The potential of HPV vaccination to significantly reduce cervical cancer incidence cannot be overstated. Schisler et al. demonstrated that the nonavalent vaccine could prevent up to 88% of HPV‐related cancers in NHPI populations, compared to just 28% preventable with earlier quadrivalent vaccines [23]. The stark contrast between the potential efficacy of vaccination and the current low uptake highlights a missed opportunity for cancer prevention. Novinson et al. and Dela Cruz (2020) illustrate that culturally tailored materials and healthcare provider recommendations can improve vaccine uptake, but these efforts require scalability and integration into broader public health strategies [18, 20].

While NHPI‐specific disparities are significant, the US as whole has fairly comparable rates of HPV vaccination as other developed nations [37]. Additionally, the US has high overall Pap screening rates when compared to many countries, but lags specifically in populations that are foreign‐born, have lower socioeconomic status, or are from certain racial or ethnic groups [38]. Future targeted endeavors focused on the NHPI population would likely benefit other communities that similarly have lower rates of vaccination and screening.

### 4.3 Cervical Cancer Treatment and Outcomes

NHPI women face profound disparities in cervical cancer treatment, from delayed care to poor survival outcomes. For example, Lee et al. found that only 22% of NHPI women received care at high‐volume facilities compared to 33% of aggregated Asian American populations [25]. Such discrepancies mirror findings in African American populations, where treatment at low‐volume facilities correlates with poorer survival [39, 40]. The delays in treatment initiation reported by Lee et al. (39% starting after 8 weeks) further compound these disparities, aligning with evidence that treatment delays directly increase mortality risk in cervical cancer patients [25].

The 5‐year survival rate of 32% reported by Robison et al. for Pacific Islanders starkly contrasts with the 71% rate for NHW women [28]. This disparity, while attributable in part to late‐stage presentation, also reflects systemic inequities in healthcare access and quality [26]. Studies in other minority groups have shown that addressing socioeconomic determinants, such as insurance coverage and geographic access, can improve outcomes. However, the intersectionality of barriers NHPI populations face—including cultural stigma, mistrust of the healthcare system, and geographic isolation—requires a more nuanced approach.

Programs like the Pacific Island Health Care Project (PIHCP), which improved adherence to standard‐of‐care radiation therapy, demonstrate that targeted healthcare delivery models can mitigate some of these disparities [26]. However, the scalability of such programs remains a challenge, particularly given the resource constraints in many Pacific Island territories.

## 5 Limitations and Future Directions

This study's limitations include high heterogeneity in meta‐analyses, small sample sizes, reliance on self‐reported data, and the predominance of cross‐sectional designs, which restrict causal inferences and generalizability. Future research should prioritize disaggregated analyses of NHPI subgroups, longitudinal studies to assess the long‐term impact of interventions, and integration of culturally tailored strategies into national public health programs. Policy advocacy should address systemic inequities, such as limited access to high‐volume care and healthcare shortages in Pacific territories, to reduce disparities and improve cervical cancer outcomes for NHPI populations.

## 6 Conclusion

NHPI populations face significant disparities in cervical cancer prevention, vaccination, and treatment, driven by systemic, cultural, and individual barriers. While culturally tailored interventions demonstrate potential, addressing these disparities requires a multifaceted approach, integrating disaggregated research, targeted policy changes, and scalable community‐based programs. These findings emphasize the urgent need for equity‐focused strategies to reduce the burden of cervical cancer in this underserved population.

## Consent

The authors have nothing to report.

## Conflicts of Interest

The authors declare no conflicts of interest.

## Synopsis

The paper reviews and analyzes disparities in cervical cancer prevention and treatment among Native Hawaiian and Pacific Islander (NHPI) populations, revealing low rates of Pap testing and HPV vaccination due to barriers like healthcare access and cultural stigma. Effective interventions include culturally tailored education and community engagement. The study emphasizes the need for culturally tailored interventions, policy changes, and further research into applicable solutions to improve cervical cancer outcomes for NHPI communities.

## Supporting information

PRISMA 2020 checklist 2 2.

## Data Availability

The data that support the findings of this study are available from the corresponding author upon reasonable request.

## References

[1] C. B. Biddell , M. C. O'Leary , S. B. Wheeler , and L. P. Spees , “Variation in Cervical Cancer Screening Preferences Among Medically Underserved Individuals in the United States: A Systematic Review,” Cancer Epidemiology, Biomarkers & Prevention 29, no. 8 (2020): 1535–1548, 10.1158/1055-9965.EPI-20-0306. PMC7415615 32457182

[2] D. X. Yang , P. R. Soulos , B. Davis , C. P. Gross , and J. B. Yu , “Impact of Widespread Cervical Cancer Screening: Number of Cancers Prevented and Changes in Race‐Specific Incidence,” American Journal of Clinical Oncology 41, no. 3 (2018): 289–294, 10.1097/COC.0000000000000264. 26808257 PMC4958036

[3] D. Singh , J. Vignat , V. Lorenzoni , et al., “Global Estimates of Incidence and Mortality of Cervical Cancer in 2020: A Baseline Analysis of the WHO Global Cervical Cancer Elimination Initiative,” Lancet Global Health 11, no. 2 (2023): e197–e206, 10.1016/S2214-109X(22)00501-0. 36528031 PMC9848409

[4] M. Arbyn , E. Weiderpass , L. Bruni , et al., “Estimates of Incidence and Mortality of Cervical Cancer in 2018: A Worldwide Analysis,” Lancet Global Health 8, no. 2 (2020): e191–e203, 10.1016/S2214-109X(19)30482-6. 31812369 PMC7025157

[5] J. Z. Shing , J. Corbin , A. R. Kreimer , et al., “Human Papillomavirus‐Associated Cancer Incidence by Disaggregated Asian American, Native Hawaiian, and Other Pacific Islander Ethnicity,” JNCI Cancer Spectrum 7, no. 2 (2023): pkad012, 10.1093/jncics/pkad012. 36790075 PMC10017119

[6] B. N. Morey , R. C. Chang , K. B. Thomas , et al., “No Equity Without Data Equity: Data Reporting Gaps for Native Hawaiians and Pacific Islanders as Structural Racism,” Journal of Health Politics, Policy and Law 47, no. 2 (2022): 159–200, 10.1215/03616878-9517177. 34522960 PMC10959240

[7] M. Mouttapa , M. Cunningham , and S. P. Tanjasiri , “Awareness of and Support for HPV Vaccination Among Pacific Islander Women in Southern California,” Journal of Cancer Education 37, no. 5 (2022): 1372–1377, 10.1007/s13187-021-01965-9. 33539008 PMC8333191

[8] O. Farajimakin , “Barriers to Cervical Cancer Screening: A Systematic Review,” Cureus 16, no. 7 (2024): 65555, 10.7759/cureus.65555. PMC11347962 39192892

[9] N. Haynes , A. Kaur , J. Swain , J. J. Joseph , and L. C. Brewer , “Community‐Based Participatory Research to Improve Cardiovascular Health Among US Racial and Ethnic Minority Groups,” Current Epidemiology Reports 9, no. 3 (2022): 212–221, 10.1007/s40471-022-00298-5. 36003088 PMC9392701

[10] J. Y. Chen , A. L. Diamant , M. Kagawa‐Singer , N. Pourat , and C. Wold , “Disaggregating Data on Asian and Pacific Islander Women to Assess Cancer Screening,” American Journal of Preventive Medicine 27, no. 2 (2004): 139–145, 10.1016/j.amepre.2004.03.013. 15261901

[11] C. C. McDaniel , H. H. Hallam , T. Cadwallader , H. Y. Lee , and C. Chou , “Persistent Racial Disparities in Cervical Cancer Screening With Pap Test,” Preventive Medicine Reports 24 (2021): 101652, 10.1016/j.pmedr.2021.101652. 34976700 PMC8684022

[12] N. Aitaoto , J. U. Tsark , D. W. Tomiyasu , B. A. Yamashita , and K. L. Braun , “Strategies to Increase Breast and Cervical Cancer Screening Among Hawaiian, Pacific Islander, and Filipina Women in Hawai'i,” Hawaii Medical Journal 68, no. 9 (2009): 215–222. 19842363 PMC4850232

[13] J. W. Weiss , M. Mouttapa , L. Sablan‐Santos , J. DeGuzman Lacsamana , L. Quitugua , and S. Park Tanjasiri , “Decision Making for Pap Testing Among Pacific Islander Women,” Health Education Research 31, no. 6 (2016): 792–802, 10.1093/her/cyw044. 27744355 PMC5141967

[14] S. P. Tanjasiri , M. Mouttapa , L. Sablan‐Santos , and L. F. Quitugua , “What Promotes Cervical Cancer Screening Among Chamorro Women in California?,” Journal of Cancer Education 27, no. 4 (2012): 725–730, 10.1007/s13187-012-0394-4. 22806217 PMC3500582

[15] M. Mouttapa , S. P. Tanjasiri , J. W. Weiss , et al., “Associations Between Women's Perception of Their Husbands'/Partners' Social Support and Pap Screening in Pacific Islander Communities,” Asia‐Pacific Journal of Public Health 28, no. 1 (2016): 61–71, 10.1177/1010539515613412. 26646422 PMC5142849

[16] S. P. Tanjasiri , M. Mouttapa , L. Sablan‐Santos , et al., “Design and Outcomes of a Community Trial to Increase Pap Testing in Pacific Islander Women,” Cancer Epidemiology, Biomarkers & Prevention 28, no. 9 (2019): 1435–1442, 10.1158/1055-9965.EPI-18-1306. PMC12626035 31186260

[17] J. H. Tran , M. Mouttapa , T. Y. Ichinose , J. K. Pang , D. Ueda , and S. P. Tanjasiri , “Sources of Information That Promote Breast and Cervical Cancer Knowledge and Screening Among Native Hawaiians in Southern California,” Journal of Cancer Education 28, no. 4 (2013): 588–594, 10.1007/s13187-010-0078-x. PMC3709576 20237883

[18] D. Novinson , M. Puckett , J. Townsend , et al., “Increasing Awareness of Gynecologic Cancer Risks and Symptoms Among Asian, Native Hawaiian and Pacific Islander Women in the US‐Associated Pacific Island Jurisdictions,” Asian Pacific Journal of Cancer Prevention: APJCP 18, no. 8 (2017): 2127–2133, 10.22034/APJCP.2017.18.8.2127. 28843233 PMC5697471

[19] S. V. Gopalani , A. E. Janitz , S. A. Martinez , J. E. Campbell , and S. Chen , “HPV Vaccine Initiation and Completion Among Native Hawaiian and Pacific Islander Adults, United States, 2014,” Asia Pacific Journal of Public Health 33, no. 5 (2021): 502–507, 10.1177/10105395211027467. 34184572 PMC8892586

[20] M. R. I. Dela Cruz , K. L. Braun , J. A. U. Tsark , C. L. Albright , and J. J. Chen , “HPV Vaccination Prevalence, Parental Barriers and Motivators to Vaccinating Children in Hawai'i,” Ethnicity & Health 25, no. 7 (2020): 982–994, 10.1080/13557858.2018.1473556. 29745749 PMC6230317

[21] C. L. Fok , M. Fifita , and S. P. Tanjasiri , “Decision‐Making Regarding Elective Child and Adolescent Vaccinations Among Native Hawaiian and Pacific Islander Parents in Orange County,” Health Promotion Practice 26, no. 1 (2025): 114–123, 10.1177/15248399231193707. 37772336 PMC11689782

[22] M. Mouttapa , M. Cunningham , and S. P. Tanjasiri , “Awareness of and Support for HPV Vaccination Among Pacific Islander Women in Southern California,” Journal of Cancer Education 37, no. 5 (2022): 1372–1377, 10.1007/s13187-021-01965-9. 33539008 PMC8333191

[23] T. M. Schisler , A. K. Bhavsar , B. P. Whitcomb , et al., “Human Papillomavirus Genotypes in Pacific Islander Cervical Cancer Patients,” Gynecologic Oncology Reports 24 (2018): 83–86, 10.1016/j.gore.2018.04.007. 29915803 PMC6003428

[24] M. R. I. Dela Cruz , J. A. U. Tsark , J. J. Chen , C. L. Albright , and K. L. Braun , “Human Papillomavirus (HPV) Vaccination Motivators, Barriers, and Brochure Preferences Among Parents in Multicultural Hawai'i: A Qualitative Study,” Journal of Cancer Education 32, no. 3 (2017): 613–621, 10.1007/s13187-016-1009-2. 26951482 PMC5014724

[25] S. S. Lee , H. T. Gold , S. C. Kwon , B. Pothuri , and M. D. S. Lightfoot , “Guideline Concordant Care for Patients With Locally Advanced Cervical Cancer by Disaggregated Asian American and Native Hawaiian/Pacific Islander Groups: A National Cancer Database Analysis,” Gynecologic Oncology 182 (2024): 132–140, 10.1016/j.ygyno.2023.12.026. 38262236

[26] C. Sitler , K. P. Bunch , D. Padro , and C. R. Miller , “Underserved Pacific Islanders With Locally Advanced Cervical Cancer Receive Higher Rates of Standard‐of‐Care Radiation Treatment Through the Pacific Island Health Care Project and Military Health System Compared to the Average U.S. Population,” Military Medicine 188, no. 3–4 (2023): e792–e796, 10.1093/milmed/usab325. 34453178

[27] F. D. V. Ho , A. Thaploo , K. Wang , et al., “Cervical Cancer Disparities in Stage at Presentation for Disaggregated Asian Americans, Native Hawaiians, and Pacific Islanders,” American Journal of Obstetrics and Gynecology 232 (2024): 310–315, 10.1016/j.ajog.2024.08.027. 39179090

[28] S. W. Robison , C. S. Dietrich , D. A. Person , and J. H. Farley , “Ethnic Differences in Survival Among Pacific Island Patients Diagnosed With Cervical Cancer,” Gynecologic Oncology 84, no. 2 (2002): 303–308, 10.1006/gyno.2001.6518. 11812091

[29] S. P. Tanjasiri , J. W. Weiss , L. Santos , et al., “CBPR‐Informed Recruitment and Retention Adaptations in a Randomized Study of Pap Testing Among Pacific Islanders in Southern California,” Progress in Community Health Partnerships: Research, Education, and Action 9, no. 3 (2015): 389–396, 10.1353/cpr.2015.0067. 26548790 PMC5142847

[30] C. C. Gotay , R. O. Banner , D. S. Matsunaga , et al., “Impact of a Culturally Appropriate Intervention on Breast and Cervical Screening Among Native Hawaiian Women,” Preventive Medicine 31, no. 5 (2000): 529–537, 10.1006/pmed.2000.0732. 11071833

[31] D. Kepka , J. Bodson , D. Lai , et al., “Factors Associated With Human Papillomavirus Vaccination Among Diverse Adolescents in a Region With Low Human Papillomavirus Vaccination Rates,” Health Equity 2, no. 1 (2018): 223–232, 10.1089/heq.2018.0028. 30283871 PMC6128445

[32] B. E. Sirovich and H. G. Welch , “The Frequency of Pap Smear Screening in the United States,” Journal of General Internal Medicine 19, no. 3 (2004): 243–250, 10.1111/j.1525-1497.2004.21107.x. 15009779 PMC1492158

[33] T. K. L. Boitano , P. Ketch , J. G. Maier , et al., “Increased Disparities Associated With Black Women and Abnormal Cervical Cancer Screening Follow‐Up,” Gynecologic Oncology Reports 42 (2022): 101041, 10.1016/j.gore.2022.101041. 35898199 PMC9309676

[34] C. M. Johnson , J. R. Sharkey , W. R. Dean , J. A. St John , and M. Castillo , “Promotoras as Research Partners to Engage Health Disparity Communities,” Journal of the Academy of Nutrition and Dietetics 113, no. 5 (2013): 638–642, 10.1016/j.jand.2012.11.014. 23375463 PMC3633728

[35] C. L. Ejezie , L. S. Savas , C. Durand , R. Shegog , and P. Cuccaro , “The Prevalence of Human Papillomavirus Vaccination Among Racial and Ethnic Minority Adolescents During the COVID‐19 Pandemic,” JNCI Cancer Spectrum 7, no. 5 (2023): pkad065, 10.1093/jncics/pkad065. 37651597 PMC10521629

[36] J. Hirth , “Disparities in HPV Vaccination Rates and HPV Prevalence in the United States: A Review of the Literature,” Human Vaccines & Immunotherapeutics 15, no. 1 (2019): 146–155, 10.1080/21645515.2018.1512453. 30148974 PMC6363146

[37] J. Han , L. Zhang , Y. Chen , et al., “Global HPV Vaccination Programs and Coverage Rates: A Systematic Review,” EClinicalMedicine 84 (2025): 103290, 10.1016/j.eclinm.2025.103290. 40547442 PMC12179740

[38] eCM eClinicalMedicine EClinicalMedicine ., “Global Strategy to Eliminate Cervical Cancer as a Public Health Problem: Are We on Track?,” EClinicalMedicine 55 (2023): 101842, 10.1016/j.eclinm.2023.101842. 36712891 PMC9874313

[39] D. A. Barrington , S. E. Dilley , E. E. Landers , et al., “Distance From a Comprehensive Cancer Center: A Proxy for Poor Cervical Cancer Outcomes?,” Gynecologic Oncology 143, no. 3 (2016): 617–621, 10.1016/j.ygyno.2016.10.004=. 27720232 PMC5116397

[40] M. Towner , J. J. Kim , M. A. Simon , D. Matei , and D. Roque , “Disparities in Gynecologic Cancer Incidence, Treatment, and Survival: A Narrative Review of Outcomes Among Black and White Women in the United States,” International Journal of Gynecological Cancer 32, no. 7 (2022): 931–938, 10.1136/ijgc-2022-003476. 35523443 PMC9509411

